# The Removal of Selected Inorganics from Municipal Membrane Bioreactor Wastewater Using UF/NF/RO Membranes for Water Reuse Application: A Pilot-Scale Study

**DOI:** 10.3390/membranes11020117

**Published:** 2021-02-06

**Authors:** Mujahid Aziz, Godwill Kasongo

**Affiliations:** Environmental Engineering Research Group (*EnvERG*), Department of Chemical Engineering, Faculty of Engineering and the Built Environment, Cape Peninsula University of Technology, Bellville, P. O. Box 1906, Cape Town 7535, South Africa; godwillkas26@gmail.com

**Keywords:** membrane bioreactor (MBR), secondary effluent, ultrafiltration (UF), inorganics, Nanofiltration (NF), reverse Osmosis (RO), chemical oxygen demand (COD), municipal wastewater treatment works (MWWTWs), flux, reuse

## Abstract

Membrane technology has advanced substantially as a preferred choice for the exclusion of widespread pollutants for reclaiming water from various treatment effluent. Currently, little information is available about Ultrafiltration (UF)/Nanofiltration (NF)/Reverse Osmosis (RO) performance at a pilot scale as a practical engineering application. In this study, the effluent from a full-scale membrane bioreactor (MBR) municipal wastewater treatment works (MWWTWs) was treated with an RO pilot plant. The aim was to evaluate the effect of operating conditions in the removal of selected inorganics as a potential indirect water reuse application. The influent pH, flux, and membrane recovery were the operating conditions varied to measure its influence on the rejection rate. MBR/RO exhibited excellent removal rates (>90%) for all selected inorganics and met the standard requirements for reuse in cooling and irrigation system applications. The UF and NF reduction of inorganics was shown to be limited to meet water standards for some of the reuse applications due to the high Electron Conductivity (EC > 250 μS·cm^−1^) levels. The MBR/NF was irrigation and cooling system compliant, while for the MBR/UF, only the cooling system was compliant.

## 1. Introduction

Water reclamation is substantial to contribute to the increasing demand for water due to climate change, population growth, and over-consumption [1]. Municipal wastewater treatment constitutes a more reliable and significant source for reclaimed water [2,3]. Membrane bioreactor (MBR) technology has drawn much attention for the treatment of municipal wastewater due to its advantages, which include a better effluent quality compared to parallel processes, absolute control of solids, and hydraulic retention times, as well as a smaller footprint [4]. However, in many cases, high-quality effluent provided for discharge by MBR systems is still not able to be used directly as irrigation and process water, because it does not meet the recommended final pollutant concentrations for reuse [5].

Membrane technology has been accepted as the most effective technique for the removal of inorganic and organic pollutants due to its outstanding performance [3]. Reverse Osmosis (RO) has been mostly used for desalination, the purification of brackish [6], seawater [7], and wastewater [8,9], due to its ability to achieve high particulate rejection levels [5]. Ultrafiltration (UF), Nanofiltration (NF) and RO are tertiary pressure-driven membrane processes that can potentially eradicate dissolved species not removed by the MBR effluent [10]. Acero et al. (2010) [2] reported that treated municipal wastewater effluent is considered a source to produce reclaimed water [11,12] and can help inhibit the harmful effects of algal blooms and eutrophication in urban water systems [13]. Numerous authors concur that the MBR process, combined with tertiary treatment, is found to be suitable for the purification of municipal wastewater to produce high-quality water for reuse [10,11,12]. Some studies concluded that a combined MBR–NF/RO system could be considered as a possible alternative for treated wastewater recycling for irrigation purposes [6,14].

Membranes were operating at pH ranges between 6 and 8, which are perfect separation conditions for conventional emerging contaminants (CEC) such as pharmaceuticals, pesticides, industrial chemicals, surfactants, and personal care products. Findings by Xu et al. (2005) [15] specified that NF and extra-low energy (XLE) RO membranes, with molecular weight cut-off (MWCO) of 200 Da and less, perform similarly to conventional RO membranes when removing CEC. The membrane surface charge in high-pressure (HP) membranes are more important for the rejection than the MWCO [15]. De Souza et al. (2020) [13] concur with Xu et al. (2005) [15] regarding the MWCO, but they reiterate that the separation mechanisms applicable on the membrane surfaces are adsorption, steric hindrance, and electrostatic effects. According to Ezugbi and Rathilal (2020) [16], membrane technology has the potential of connecting the reliability and economic gap due to its accessibility and environmental sustainability.

In this study, the performances of three different membranes, namely Ultrafiltration (UF), Nanofiltration (NF), and Reverse Osmosis (RO), were evaluated in the removal of inorganic compounds and chemical oxygen demand (COD) from secondary municipal sewage wastewater treatment plant (MSWWTP) MBR effluent. The objective of this study was to assess the effects of operating condition parameters, such as pH, permeate flux, and system recovery, for reuse application. The reuse of a secondary MSWWTP MBR effluent for cooling system application and agricultural irrigation could increase agricultural production as well as water availability. The reduction of over-abstraction of surface and groundwater due to integrated usage of water resources will decrease water scarcity.

## 2. Materials and Methods

### 2.1. Full-Scale MBR and RO Pilot-Plant Hybrid System

**Figure 1:**
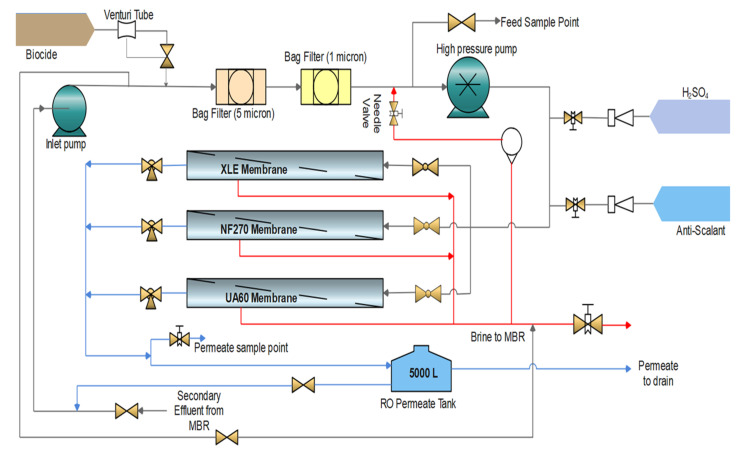
Process flow diagram of the Ultrafiltration (UF)/Nanofiltration (NF)/Reverse Osmosis (RO) pilot plant.

The wastewater treatment works (WWTWs) is equipped to treat the wastewater from the largest informal settlement and its surroundings in the province. It is situated in the City of Cape Town in the Western Cape, South Africa. The MBR system incorporates ZeeWeed^®^ 500 ultrafiltration membranes (GE Zenon, trading as Suez Technologies and Solution, Trevose, PA, USA), producing 18 megaliters of effluent per day. The pilot plant consisted of three different thin film composite (TFC) polyamide (PA) membrane modules, in parallel, which was subjected to various experimental running conditions (Table 1). The secondary MBR effluent (Table 2) was used to feed into the UF/NF/RO pilot plant (Figure 1). Batches, 8 h, once through experimental mode runs, were conducted with individual membranes at any given time.

A frequency converter controlled the influent flow rate and the operational pressure through the inlet and high-pressure pumps. Two bag filters with pore sizes of 5.0 and 1.0 μm were installed between these two pumps to prevent potential damage to the membranes by large particles. The pressures, flow rates, and temperature of influent, effluent, and brine were all monitored by online pressure gauges, rotameters, and thermometers. Online monitoring instruments measured the pH and conductivity of influent, effluent, and brine. Two online automatic dosing systems for pH and antiscalant are included. Phosphonic acid (H_2_O_3_P^+^) Vitec 3000, which is a broad spectrum antiscalant and dispersant liquid obtained from Avista Technologies, Inc., South Africa, was used to minimize fouling.

The feedwater pH fluctuation (6.5 and 7) affected the membrane surface charge and the state of the solute, rendering the rejection of pollutants complex. The experimental tests were conducted with both constant pH and uncontrolled pH to describe the effects on salt rejection and the removal of inorganics and COD. Sulfuric acid (H_2_SO_4_) was used to keep the pH constant at 6.5.

The RO system was also equipped with an online membrane cleaning system by flushing an industrial biocide; Hydrex 7000, obtained from Veolia Water Technologies (Pty Ltd.), Paarl, South Africa; daily after an operation.

### 2.2. UF/NF/RO Membranes

Pilot plant experimental runs were carried out with three commercial spiral wound membranes: (1) XLE, a polyamide extra low energy RO membrane from DOW-Filmtec (Midland, MI, USA), with an MWCO of approximately 200 Da [17]; (2) NF270, a polyamide loose NF membrane from DOW-Filmtec (Midland, MI, USA), with an MWCO of approximately 400 Da [17]; and (3) UA60, a piperazine loose UF membrane from TriSep (Goleta, CA, USA) with an MWCO of approximately 1000 Da [17]. These membranes were chosen because they have MWCOs covering the MW range (200–1000 Da) of most inorganics reported in the literature [16]. The characteristics of these membranes are presented in Table 3.

### 2.3. Membrane Energy Consumptions

The energy usage of membranes systems contributes to nearly 35–45% of the total permeate production cost [19,20]. Therefore, it is imperative to evaluate the energy consumption of membranes and study the effects of operating conditions such as percentage recovery and flux on the consumption of energy. The sources of energy consumption of membranes systems include the feedwater intake and pre-treatment; high-pressure pumps; membrane type; and post-treatment [19]. The principal source of energy consumption is the high-pressure pump, which is essential to drive water flux across the membrane [21]. The pump energy usage can be expressed as specific energy consumption (SEC), which can be obtained using the following equation [20]:

(1) $$SEC=\frac{\Delta P}{\gamma }=\frac{\Delta P\times Q_{F}}{Q_{P}}$$

where ∆P is the transmembrane pressure (Pa), ϒ is the percentage recovery, Q_F_ is the feed flowrate, and Q_P_ the permeate flow rate.

### 2.4. Analytical Methods and Water Analysis

The influent, effluent, and brine were all collected to investigate the operation performance of the RO system. The physicochemical parameters assessed were Electrical Conductivity (EC), Total Dissolved Solids (TDS), pH, Temperature (T), Turbidity, Ammonium (NH_4_^2+^), Phosphate (PO_4_), Nitrate (NO_3_), and Chemical Oxygen Demand (COD). Sampling was carried out from four different sampling points: (1) MBR effluent; (2) permeate of UF; (3) permeate of NF; (4) permeate of RO element. To avoid frequent fluctuations in concentrations, each sample taken from the pilot plant was an 8-h composite sample taken for the duration of each experimental run. All water samples were collected in amber glass bottles (2.5 L) prewashed with nitric acid and rinsed thoroughly with distilled water. Samples were filtered through 1.0 μm pore size glass fiber filter paper (Whatman GF/B); then, the filtrates were stored at 4 °C and analyzed within 24 h of collection. All equipment and meters for the on-site measurements were calibrated and checked according to the manufacturer’s instruction. EC, T, and TDS were measured using a Crison CM 35+ handheld meter (Merck Pty Ltd., Bellville, Cape Town, South Africa). The pH measured with Jenway 3510 Bench pH/mV Meter and Turbidity with an HF Scientific Micro TPI Infrared Turbidity Meter. COD samples were digested in a Thermo reactor Model HI839800-02 (Hanna Pty Ltd., Bellville, Cape Town, South Africa) and measured using a COD Meter and Multiparameter photometer Model HI83214-02 (Hanna Pty Ltd., Bellville, Cape Town, South Africa). The concentration levels of NH_4_^2+^, PO_4_, and NO_3_ (Hanna Pty Ltd., Bellville, Cape Town, South Africa) were analyzed using the Multiparameter photometer Model HI83214-02 according to the Standard Methods.

### 2.5. Statistical Analysis

The data presented were analyzed with statistical calculations to approve the significance of the data obtained. Analyses of variance (ANOVA) and T-test with a significance level of 0.05 were applied to evaluate correlations between membrane type (UF, NF, and RO) and pH (controlled and uncontrolled), respectively.

## 3. Results and Discussion

### 3.1. Salt Rejection and Total Dissolved Solids (TDS)

**Figure 2:**
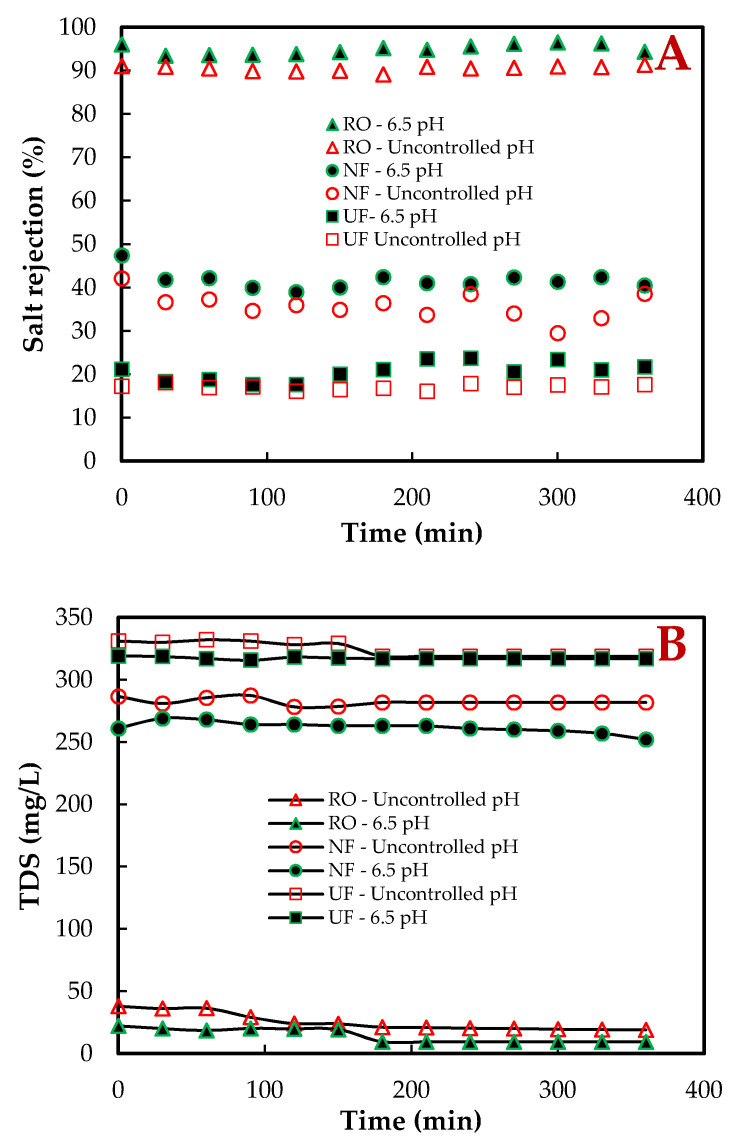
The measured effluents of the UF, NF, and RO membrane treatment at uncontrolled and controlled 6.5 pH pilot plant condition. Salt rejection (**A**) and TDS (**B**), all at a flux of 30 L·m^−2^h^−1^, and 75% recovery.

The performance of membranes was assessed by measuring the physicochemical parameters, salt rejection and total dissolved solids (TDS), with the pilot plant operation condition of flux, recovery at 30 L·m^−2^h^−1^, 75% respectively, as well as control and uncontrolled pH. Figure 2 shows the permeate salt rejection (Figure 2A) and TDS (Figure 2B) as a function of time, obtained for all three membranes (UF/NF/RO) during experimental runs on the pilot plant. The RO (XLE) membrane rejection was the highest between 94.4–96.6% at controlled (6.5) and 89.2–91.4%, uncontrolled pH. The UF (UA60) and NF (NF270) membranes performed as expected and had better rejection at a controlled pH. Although there was MBR effluent (real feed) with concentration variation into the UF/NF/RO pilot plant, as can be seen with the TDS, the results still indicate that the performance of the membranes was stable throughout the experimental study. The RO salt rejection usually is high due to its membrane design characteristics, where the skin layer is much denser than the other two, UF and NF. The mechanisms that can be attributed to the rejection of ionic species in the water are size exclusion, charge, and ionic electrostatic interactions of the ions with the surface of the membrane. It is reported that monovalent ions in the feed water can generally pass through the membrane more easily than divalent ions due to size exclusion [18]. The UF and NF membranes have similar separation characteristics; however, the membrane parameters are quite different.

Garcia-Aleman et al. (2004) [22] state that the transport and selectivity of NF membranes are mainly due to steric/hydrodynamic effects and charge repulsion. The relative size of the ions causes the steric effect to the membrane pores, and the repulsion effect is caused by the charged nature of the membrane and electrolytes. The NF270 is a loose NF membrane, but it is tighter than the UF, with relatively high permeability and charge density. The UA60 membrane has a larger pore radius, is less permeable, and has a higher surface charge density; thus, steric effects are not as applicable. During salt separation, the UA60 membrane depends exclusively on Donnan exclusion [22]. According to Üstün et al. (2011) [23], the UF and NF membrane surfaces are negatively charged at pH values higher than 4. The pH of the MBR-UF/NF/RO influent in this study was between 6 and 7, thus presenting a negative charge density on the membrane surface. The primary mechanism of ion rejection by these (UF and NF) membranes is the sieving mechanism [24]. A solution–diffusion model describes the XLE membrane transport mechanism because of the nominal pore size, where diffusion dominates over convection [25].

### 3.2. Chemical Oxygen Demand (COD)

**Figure 3:**
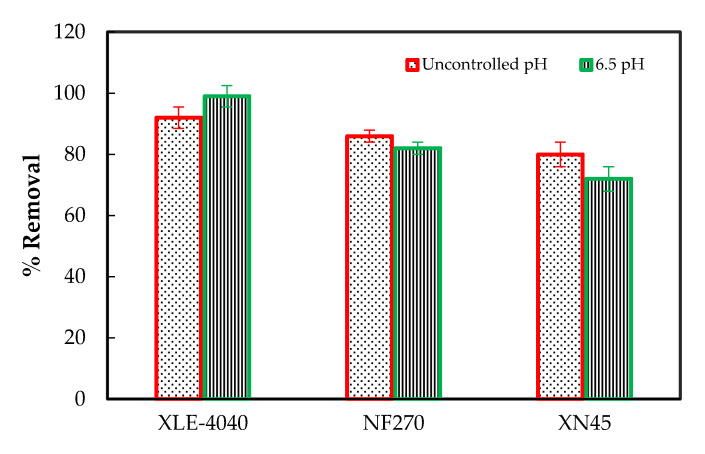
The measured effluents of the UF, NF, and RO membrane treatment at uncontrolled and controlled 6.5 pH pilot plant condition of chemical oxygen demand (COD) removal.

The pH level of water defines its application for different purposes. Low or high pH has a poisonous effect on marine life and alters the solubility of other chemical pollutants and elements in the water. The South African limit for pH in the water for reuse is 6 to 9 [17]. Chemical oxygen demand is described as the amount of strong oxidant required to break down both organic and inorganic substances in water. The removal of COD with all membranes is presented in Figure 3, where the RO percentage removal of 92% and 99% for uncontrolled and controlled pH, respectively, were significantly higher than the UF and NF membranes (*p* = 0.018 for UF, *p* = 0.013 for NF and *p* = 0.009 for RO; α = 0.05). This is consistent with a similar study of MBR/NF and MBR/RO membrane effluent rejection [26]. MBR is considered a relatively improved treatment process for the exclusions of COD compared to conventional activated sludge processes as a pre-treatment for NF and RO reuse [10].

The effect of pH on the COD removal with the NF and UF membranes appeared to have the opposite effect as compared to the RO membrane, where the higher percentage was achieved. The pH range with uncontrolled pH experimental runs was between 6.7 and 7.1, while experimental runs with controlled pH maintained the latter at 6.5. The COD removal has been reported to increase with increasing the pH, which was in part attributed to the rise in the hydroxide ions concentration, increasing the production of hydroxyl free radicals [21]. Therefore, this may suggest that the increase in pH was a predominant factor in the removal of COD when using the UF and NF membranes. Other researchers have reported changes in properties such as pH to affect contaminant removal, which was found to be substantially lower when operated without pH control [22]. The COD percentage removals for UF were 80 and 72; NF were 85 and 82. This is in the range of a study of Xu et al. (2020) [27] where the NF membrane showed a COD percentage removal of 90. The best removal of COD achieved with a controlled pH when using the RO membrane may be explained by the fact that a controlled pH results in a higher and more sustained osmotic flow, which caused a more significant COD removal [28], as well as the surface of the membrane, which became less negative with the decrease in pH as compared to experimental with no adjustment of pH [29].

### 3.3. Inorganics Removal

**Figure 4:**
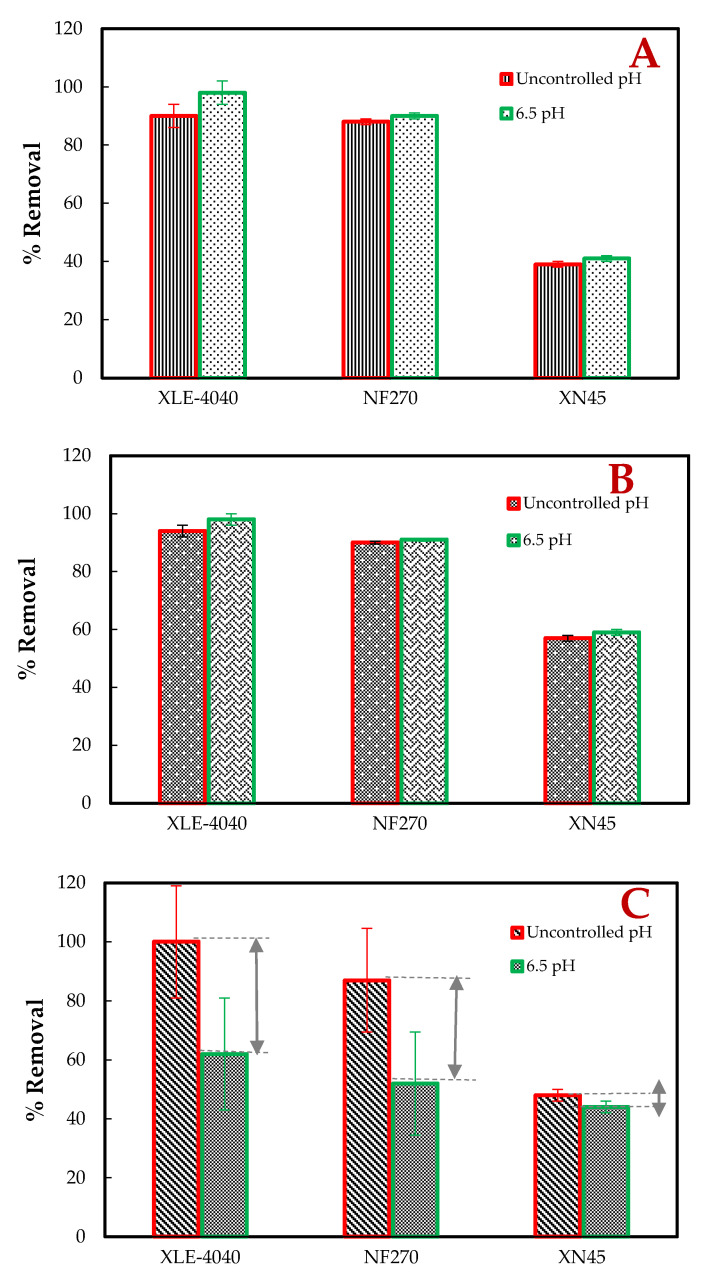
The measured effluents of the UF, NF, and RO membrane treatment at uncontrolled and controlled 6.5 pH pilot plant condition. Phosphate (**A**), Phosphorous (**B**), and Ammonia removal (**C**).

The permeate quality of the MBR/UA60, MBR/NF270 and MBR/XLE units of the selected inorganics are summarized in Figure 4. Shad et al. (2019) [30] confirmed that inorganics are found in municipal wastewater originating from domestic and industrial products such as pesticides, preservatives, surfactants, perfluorochemicals, pharmaceutical residues, and steroidal hormones, which are all found in excreted human waste. These salts were selected to evaluate the correlation of anionic, neutral, and cationic solutes with the membrane-type and pH. It can be seen in Figure 4A and B the percentage removal of phosphate: 40, 89 and 94% and phosphorous: 58, 90.5, and 96% with the UA60, NF270, and XLE, respectively.

There was a significant difference in the removal of selected inorganics observed with the three membranes (*p* = 0.001 for uncontrolled pH, *p* = 0.043 for 6.5 pH at α = 0.05). The phosphorus removal is visibly higher than the phosphate, which is due to the size exclusion and chemical charge. Phosphorous is a neutral molecule that differs from phosphate, which is a multivalent anion that may increase electrostatic repulsion with the surface of the membrane [31]. The reduction of both phosphate and phosphorous with pH change indicates that pH adjustment affects the removal of these physicochemical properties slightly (*p* > 0.05 for both phosphorous and phosphate). Contrary, the adjustment of pH had a significant effect on ammonia percentage removal with the NF270 and XLE membranes (*p* = 0.018 at α = 0.05). This ammonia percentage removal is shown in Figure 4C, where it increased from 62 to 99% and 52 to 87%, respectively, when changing from 6.5 pH to uncontrolled. This could be explained by the fact that the pH adjustment (pH 6.5) shifted the equilibrium of ammonia, resulting in higher removal and permeance of cations than the anions due to the deprotonated carboxylic groups of the polyamide membrane [32]. According to Chu et al. (2017) and Pagès et al. (2017) [31,32], ionizable functional groups can affect water and solute permeation due to the production of pH-dependent charges on the active membrane layer. Sert et al. (2017) [8] reiterated that the higher rejection of these monovalent ions by the XLE membrane is due to its dense surface layer without pores. The UA60 and NF270 with higher MWCO are classified as loose membranes that reject monovalent ions with lower percentages by electrostatic interaction mechanism.

### 3.4. Membranes Energy Consumption Comparison and Effect of RO Operating Conditions

Energy usage of the membrane system was calculated using the specific energy consumption of pumps (SEC), as 50 to 75% of the energy consumed by RO systems emanates from the high-pressure pumps of the system [19]. Table 4 shows the SEC of the RO membranes at different conditions and compared to the UF and NF SEC calculated. The RO membrane used in this study indicated a low consumption of energy compared to other studies conducted, where a minimum SEC of 1.37 to 2 kWh·m^−3^ could be obtained at 50% recovery [20,33].

Furthermore, it shows that a change in percentage recovery has more effect on the SEC than water flux change when comparing the different experimental conditions of the RO membrane. At a lower recovery of water (50%), the SEC required was higher than the SEC at 75% recovery. This can be explained by the decline in differential pressure drop across the membrane as a function increased velocity in the concentrate stream [20]. When comparing SEC of membranes, results show that the RO membrane consumed more energy than NF membranes, and the latter consumed more than the UF membrane. This is again due to the membranes’ design characteristics difference; hence, the RO required more pressure to drive the solvent across the dense surface of the membrane.

**Figure 5:**
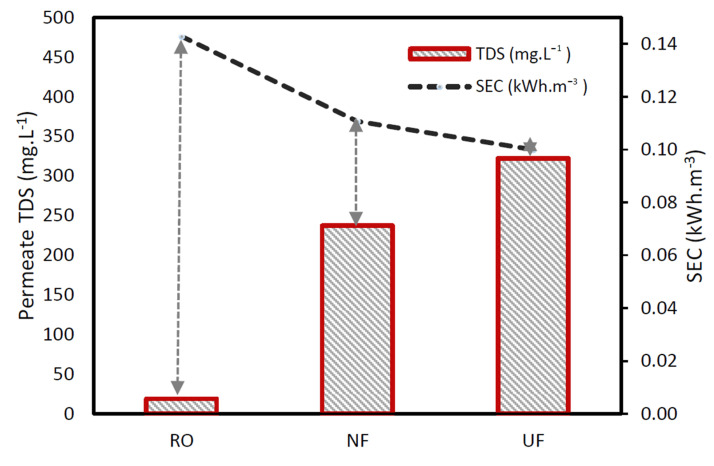
Comparison of membranes systems specific energy consumption concerning the permeate total dissolved solids (TDS) at a fixed permeate flux of 30 L·m^−2^h^−1^ and recovery of 75%.

The SEC difference between the NF and the UF is not significant, as the two membranes have similar separation characteristics. However, the energy consumption only increased by a factor of 1.42 when shifting from UF or NF to RO. No energy recovery device (ERD) was used when using the RO membrane. Therefore, the use of an ERD would help sensibly reduce the energy consumption of the RO, as suggested by several researchers [19,20,21]. It is indicated that ERD can help reduce the SEC up to 16% [19]. Figure 5 shows the specific energy consumed versus permeate total dissolved solids (TDS). In order to achieve a lower, permeate TDS concentration, more energy was required from the pump. The low TDS concentration is directly proportional to the characteristic of the membranes, which in this case required more energy usage when moving from UF to NF and RO, respectively.

### 3.5. Reuse Application for Wastewater Effluent

The effluent samples of the MBR/UF, MBR/NF, and MBR/RO were compared to the water quality requirements for reuse in cooling and irrigation applications, as summarized in Table 5. This table shows essential changes in the average inorganic and COD concentration. The MBR/RO effluent satisfied all reuse conditions required for cooling and irrigation application, and this is consistent with similar studies [23,24,33,34]. The physiochemical properties of the MBR/NF effluent are suitable for reuse in industrial cooling applications. However, they may be restricted to specific irrigation applications, because some of the parameters such as the EC (355 μS·cm^−1^) are outside the required range (<250 μS·cm^−1^) for unrestricted irrigation water quality. As expected, the MBR/UF effluent with an EC of 471 μS·cm^−1^ is only suitable for cooling system reuse application. Falizi et al. (2018) [26] cautions that water salinity (measured by EC) is the primary factor threatening crop productivity and quality with the usage of irrigation water at a pH between 6 and 9. The earth may appear wet, but if the EC is high, then the available water to the vegetations will be less. The acceptable EC limits for effluent discharge and domestic water supply usage, according to South African guidelines, are 250 μS·cm^−1^ and 70 μS·cm^−1^, respectively [26].

The results in Table 5 show that the removal of phosphate, phosphorous, and ammonia with MBR/NF270 and MBR/XLE membranes is within the specification guidelines for cooling and irrigation applications. The rejection of the phosphates and ammonia with the XLE membrane is due to size exclusion but with the UA60 and NF270 (both negatively charged membranes) charge effects. Van Voorthuizen et al. (2005) [25] explained that the difference in rejection between ammonia and phosphate for the negatively charged membranes (UA60 and NF270) could be explained by the hydrogen–phosphate ion (HPO_4_^2+^), which is bigger than the bicarbonate ion (HCO^3+^). The hydrogen–phosphate ion has a larger negative charge and will repel much more assertively with the UA60 and NF270 membrane. NH_4_^+^ cation enters and is retained by membrane pores when the hydration energy of 407 kJ·mol^−1^ and the ionic radius of 0.095, due to surface forces [36].

### 3.6. Effect of Operation Conditions on RO Membrane Rejection

#### 3.6.1. Selected Inorganic Rejection

The properties of operating conditions such as pH, permeate flux and system percentage recovery were evaluated using the RO (XLE) membrane. The parameters in the effluent are lower than those obtained in the influent, as shown in Table 6. For the experimental runs conducted at a constant pH of 6.5, the best results were attained at a permeate flux of 25 L/m^2^·hr and a system percentage recovery of 75. The highest average percentage reductions obtained for ammonia, nitrate, nitrite, phosphate and phosphorous were 98%, 100%, 83%, 97%, and 98%, respectively. Although the findings did not match expectations suggesting a slight decrease in permeate inorganics concentration when increasing flux, on the assumption that ions leakage across the membrane remains reasonably constant [30], the phenomenon may be explained by the increase in percentage recovery (75%), which allows for the mass of ions at the surface of the membrane to be blended with more permeate, resulting in a lower concentration of inorganics in the permeate.

The percentage reduction of the different inorganics tested, increase from nitrite, nitrate, phosphate, ammonia to phosphorous removal. Higher rejection of multivalent ions can be explained by the size of multivalent ions, which is larger than monovalent ones. Therefore, an increase in ion charge causes an increase in electrostatic interactions with membranes, which determine the contaminant removal mechanism [33]. The lower rejection of nitrate compared with the ammonia rejection measured with the same membranes at the same operating conditions is notable. NH_4_^+^ is regarded as a single-charged trace cation that was better rejected than the anions (NO_3_^−^) [32]. Nitrate rejection for the RO membrane follows the behavior described by Donnan exclusion theory where the co-ions (NO_3_^−^) are rejected due to electrostatic repulsion. In some studies, the nitrates in the effluent (permeate) increases. This may be due to the oxidation of ammonia and nitrite to nitrate by nitrite-oxidizing bacteria [37].

#### 3.6.2. Chemical Oxygen Demand (COD) rejection

**Figure 6:**
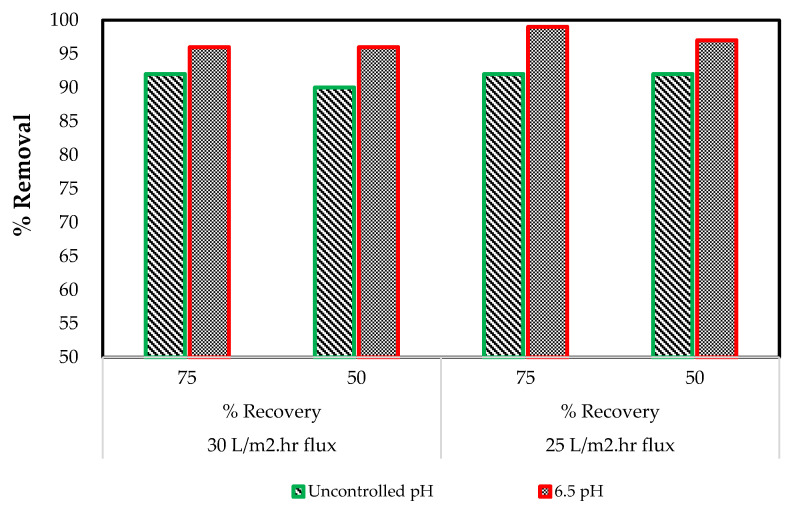
The measured effluents of the RO membrane treatment at uncontrolled and controlled 6.5 pH pilot plant conditions of COD removal.

The effects of several operating conditions such as pH, permeate flux and system percentage recovery was evaluated using the RO (XLE) membrane with the removal of COD. Figure 6 describes the results where the best COD percentage removal of 99 was obtained when the system operated at 75% recovery and 25 L·m^−2^ h^−1^ of flux, with a controlled pH of 6.5. The change in operating conditions of flux and system recovery does not have a significant effect on COD rejection, except for pH. The average COD rejection increases by 5% for a pH variable with a minimum of 96 and a maximum of 99%, respectively. This suggests that the accumulation of organic matter in the treatment of the effluent with an RO membrane can be effectively reduced with a controlled pH and by adjusting the flux and recovery. According to Paugum et al. (2004) [36], the degree of membrane ionization is a function of the effluent pH where the isoelectric point corresponds to the pH value for which the electric charge of the fixed cations neutralizes that of the anions.

#### 3.6.3. Water Turbidity Using the RO System

**Figure 7:**
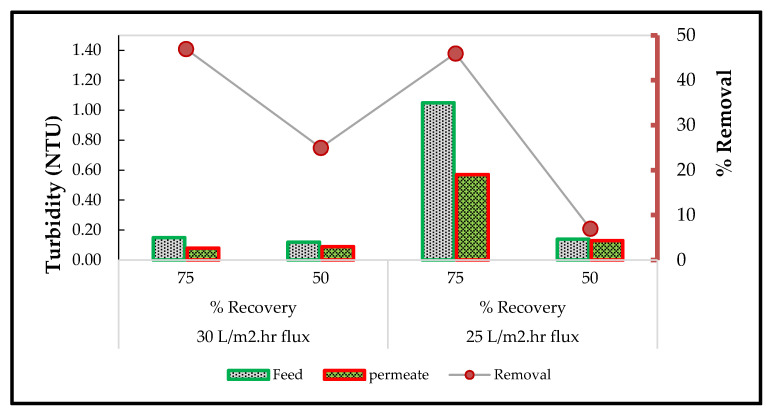
Turbidity removal using RO membrane with controlled pH at 6.5.

The effects of several operating conditions such as pH, permeate flux and system percentage recovery were evaluated using the RO (XLE) membrane with the measuring of turbidity. Figure 7 describes the results for the lowest turbidity obtained at a flux of 25 L·m^−2^ h^−1^ of flux, with a controlled pH of 6.5 and system recovery of 75% (0.08 NTU) and 50% (0.09 NTU), respectively.

The highest turbidity measurement in the permeate (0.57 NTU) was obtained at 50% recovery and 25 L·m^−2^ h^−1^. The high turbidity observed in the samples indicates the presence of finely divided organic and inorganic matter, soluble colored organic compounds and microscopic organisms. Studies have shown that too much turbidity in water can lead to interference with water treatment techniques and increase the cost.

When turbid water is chlorinated, then, a possible rise in trihalomethane (THM) precursor formation is possible [37]. All the turbidity influent or effluent results are below 1 NTU except for one influent (25 L·m^−2^ h^−1^ permeate flux, 75% recovery) at 1.05 NTU. The slightly high permeate turbidity of 0.57 NTU obtained at these operating conditions is explained by the high turbidity (1.05) of the RO influent, which influenced the turbidity of the effluent. There is no South African guideline for turbidity in effluent discharge, although the South African Target Water Quality Range for turbidity in water for domestic water supply is 0–1 NTU, while the World Health Organization (WHO) standard is 5 NTU [38].

## 4. Conclusions

In this study, the performance of UF/NF/RO technology for treating municipal MBR wastewater on a pilot scale was investigated. RO (XLE) and NF (NF270) membranes exhibited exceptional removal rates of 90%, (UF > 40%) for COD, NH_4_^2+^, PO_4_, and NO_3_. The influence of pH, permeate flux, and percentage recovery had a visible effect on the rejection of these selected inorganics. The XLE membrane showed a 99% COD rejection at operational conditions where the pH, flux, and recovery were 6.5, 25 L·m^−2^h^−1^ and 75%, respectively. Although the results show that the removal performance of inorganics and COD are significantly better with the XLE membrane, the energy consumption, however, increased by a factor of 1.42 than with UA60 or NF270 membranes. The MBR/RO comply with the standard requirements for potable and non-potable reuse applications.

## Figures and Tables

**Table 1:** Pilot plant operating conditions [8].

Parameters	Operating Conditions		
Membrane module	XLE	NF270	UA60
Recovery (%)	50; 75	75	75
Flux (L·m^−2^h^−1^)	25; 30	30	30
pH	uncontrolled; 6.5	uncontrolled	uncontrolled

**Table 2:** The physicochemical characteristics of the membrane bioreactor (MBR) effluent.

Parameter	Units	Average MBR Effluent	Limit ^1^
Electron conductivity (EC)	mS/m	56	75 ^1^
pH		6.5	5.5–9.5 ^1^
Total Dissolved Solids (TDS)	mg/L	360	450 ^1^
Chemical oxygen demand (COD)	mg/L	<20	75 ^1^
Ammonium (NH_4_^2−^)	mg/L	<0.4	3.0 ^1^
Phosphate (PO_4_)	mg/L	2.6	10 ^1^
Nitrate (NO_3_)	mg/L	13	15 ^1^
Turbidity	NTU	0.25	-
Temperature	°C	25	35 ^1^

**^1^** Department of Water and Forestry (DWAF) 2010 guideline [9].

**Table 3:** Properties of three membrane modules.

Membrane Component ^1^	Texture ^1^	Type ^1^	Rejection ^1^ %	Effective Area ^1^ (m^2^)	MWCO ^1^ (Da)	Maximum Pressure ^1^ (bar)	Maximum ^1^ Temperature (°C)	Maximum Permeate Flowrate ^1^ (m^3^/h)
RO	TFC Polyamide	Filmtec XLE−4040	99% NaCl	8.1	<200	6.9	45	9.8
NF	TFC Polyamide	Filmtec NF270-4040	>97% MgSO4	7.6	400	4.8	45	9.5
UF	TFC Piperazine	TriSep 4040-UA60-TSA	80% MgSO4	8.2	1000	7.6	45	11.4

**^1^** Obtained from the literature [18].

**Table 4:** Energy consumption comparison of RO membrane operating conditions with NF and UF membranes.

	RO				NF	UF
	30 L·m^−2^ h^−1^ Flux		25 L·m^−2^ h^−1^ Flux		30 L·m^−2^ h^−1^ Flux	
	% Recovery (ϒ)				75% Recovery (ϒ)	
Parameter	75	50	75	50		
∆P (kPa)	384.2	423.7	385.7	389.2	298.7	269.7
Q_B_ (m^3^·h^−1^)	0.081	0.243	0.068	0.204	0.063	0.205
Q_P_ (m^3^·h^−1^)	0.243	0.243	0.2024	0.204	0.19	0.205
Q_F_ (m^3^·h^−1^)	0.324	0.486	0.270	0.405	0.253	0.41
SEC (kWh·m^−3^)	0.142	0.235	0.143	0.216	0.111	0.100

**Table 5:** Characteristics of UF/NF/RO effluent average water quality with reuse criteria for wastewater in different applications.

Parameter	Irrigation [23,24]	Cooling System [34,35]	UF	NF	RO
COD (mg·L^−1^)	<50	<30	16	10	2
NH_3_ (mg·L^−1^)	<6.08	<1	0.62	0.28	0.17
P (mg·L^−1^)	<1.5	-	1.8	0.79	0.21
PO_4_ (mg·L^−1^)	<2	<7	2.07	0.91	0.45
TDS (mg·L^−1^)	<200	-	300	255	19
pH	6.5–8.4	6.8–7.2	6.5–7.05		
EC (μS·cm^−1^)	<250	<1445	471	355	37
Turbidity (NTU)	<2	<36	-	-	0.08

**Table 6:** Selected inorganics percentage removal using RO membrane.

Operating Conditions		30 L/m^2^·h Flux		25 L/m^2^·h Flux	
		% Recovery			
Water pH	Inorganic	75	50	75	50
6.5	NH_3_	92	97	98	97
	NO_3_	100	87	80	100
	NO_2_	63	60	83	82
	PO_4_	90	97	90	97
	P	92	97	98	97
Uncontrolled	NH_3_	80	87	94	92
	NO_3_	68	76	63	63
	NO_2_	61	55	86	71
	PO_4_	98	86	98	88
	P	80	87	94	92
